# Genome-Wide Association Study of Young-Onset Hypertension in the Han Chinese Population of Taiwan

**DOI:** 10.1371/journal.pone.0005459

**Published:** 2009-05-07

**Authors:** Hsin-Chou Yang, Yu-Jen Liang, Yi-Lin Wu, Chia-Min Chung, Kuang-Mao Chiang, Hung-Yun Ho, Chih-Tai Ting, Tsung-Hsien Lin, Sheng-Hsiung Sheu, Wei-Chuan Tsai, Jyh-Hong Chen, Hsin-Bang Leu, Wei-Hsian Yin, Ting-Yu Chiu, Chin-Iuan Chen, Cathy S. J. Fann, Jer-Yuarn Wu, Teng-Nan Lin, Shing-Jong Lin, Yuan-Tsong Chen, Jaw-Wen Chen, Wen-Harn Pan

**Affiliations:** 1 Institute of Statistical Science, Academia Sinica, Taipei, Taiwan; 2 Institute of Biomedical Sciences, Academia Sinica, Taipei, Taiwan; 3 Taichung Veterans General Hospital, Taichung, Taiwan; 4 Kaohsiung Medical University Chung-Ho Memorial Hospital, Kaohsiung, Taiwan; 5 National Cheng Kung University Hospital, Tainan, Taiwan; 6 National Yang-Ming University School of Medicine and Taipei Veterans General Hospital, Taipei, Taiwan; 7 Cheng Hsin Rehabilitation Medical Center, Taipei, Taiwan; 8 Min Sheng General Hospital, Taoyuan, Taiwan; University of Michigan, United States of America

## Abstract

Young-onset hypertension has a stronger genetic component than late-onset counterpart; thus, the identification of genes related to its susceptibility is a critical issue for the prevention and management of this disease. We carried out a two-stage association scan to map young-onset hypertension susceptibility genes. The first-stage analysis, a genome-wide association study, analyzed 175 matched case-control pairs; the second-stage analysis, a confirmatory association study, verified the results at the first stage based on a total of 1,008 patients and 1,008 controls. Single-locus association tests, multilocus association tests and pair-wise gene-gene interaction tests were performed to identify young-onset hypertension susceptibility genes. After considering stringent adjustments of multiple testing, gene annotation and single-nucleotide polymorphism (SNP) quality, four SNPs from two SNP triplets with strong association signals (−log_10_(p)>7) and 13 SNPs from 8 interactive SNP pairs with strong interactive signals (−log_10_(p)>8) were carefully re-examined. The confirmatory study verified the association for a SNP quartet 219 kb and 495 kb downstream of *LOC344371* (a hypothetical gene) and *RASGRP3* on chromosome 2p22.3, respectively. The latter has been implicated in the abnormal vascular responsiveness to endothelin-1 and angiotensin II in diabetic-hypertensive rats. Intrinsic synergy involving *IMPG1* on chromosome 6q14.2-q15 was also verified. *IMPG1* encodes interphotoreceptor matrix proteoglycan 1 which has cation binding capacity. The genes are novel hypertension targets identified in this first genome-wide hypertension association study of the Han Chinese population.

## Introduction

Hypertension is a common disorder that is prevalent in most populations, especially in highly industrialized regions [1]. The prevention and management of hypertension has become an important public health issue in the world. The identification of hypertension susceptibility genes and an understanding of the hypertension genetic mechanism may contribute to the development of genetic prevention, counseling and treatment for hypertension in the future. Efforts to identify hypertension genes have been ongoing for several decades [2]. Some susceptibility genes have been located using different mapping strategies. One of the mapping strategies is “candidate-gene linkage analysis”. This method is effective for mapping genes with large phenotypic effects that follow Mendelian laws of inheritance using a large-pedigree linkage approach, and its success in identifying novel hypertension genes is best described by Lifton's works [3], [4]. Using this approach, approximately 10 genes were linked to the causality of hypertension, which account for only a small fraction of the essential hypertension etiology. Another mapping strategy is “genome-wide linkage approach”. This method, which uses hundreds to thousands of short tandem-repeat polymorphisms and a large number of families, has been used in various studies that suggested multiple potential locations of hypertension genes for further research; however, the indicated regions of interest are often too broad and are not consistent across multiple studies [5]. Very few studies have fine-mapped the genes, not to mention carrying out cross-verification of these genes [6].

Much hope has thus been placed on the state-of-the-art genome-wide association study approach using a large number of dense single-nucleotide polymorphism (SNP) markers. From 2006 to 2008, several dozen large-scale genome-wide association studies were published tackling various complex diseases [7]. To date, there have been only two large-scale genome-wide association studies on hypertension, both of which were carried out by the Wellcome Trust Case Control Consortium (WTCCC) [8], [9]. Neither yielded apparent variants at the initial stage of data analysis. The Family Blood Pressure Program tried to replicate the top six SNPs identified by the WTCCC but failed to do so [10]. These frustrating findings of the above attempts underscore the need for stringent phenotype definition and powerful statistical gene mapping methods in genetic analyses of hypertension.

To increase the genetic contribution and homogeneity of the study trait, here we focus on young-onset hypertension (YOH), which has a stronger genetic component than its older counterpart [11]. Although clinical profile and candidate gene studies have sketched a blueprint for genetic susceptibility in YOH in the Han Chinese population [12]–[15], meticulous dissection of YOH by a systematic genome-wide association study has not been performed. This study aims to identify YOH susceptibility genes for the Han Chinese population based on a two-stage study design consisting of a genome-wide association study (GWAS) and a confirmatory association study (CMAS).

## Materials and Methods

### Study design and samples

We performed a two-stage case-control association scan, consisting of a GWAS for the first stage and a CMAS for the second stage, to identify YOH susceptibility genes. We obtained complete genotypic and phenotypic data from 1,008 YOH individuals and established immortalized cell lines from their lymphocytes for the Academia Sinica Multi-Center YOH Genetic Study. In addition, we also obtained genotypic and phenotypic data from 1,008 normal controls from three projects: the Taiwan Han Chinese Cell and Genome Bank [16], the Cardiovascular Disease Risk Factor Two-Township Study [17] and the Nutrition and Health Survey in Taiwan [18]. This study was approved by the Internal Review Board of Academia Sinica. A written informed consent was signed by every participant at his/her initial clinic visit. All individuals in this study were Han Chinese.

In the first-stage association mapping, GWAS, 175 YOH patients with normal body mass indices (<23 kg/m^2^), triglyceride levels (<150 mg/dl) and high density−lipoprotein cholesterol levels (>40 mg/dl) were analyzed. A one-to-one match strategy for age (±5 years) and sex was applied to select controls (n = 175) from the Taiwan Han Chinese Cell and Genome Bank [16]. In the second-stage association mapping, CMAS, a group-match strategy balancing three age groups (20–32, 32–44, 44+) and two gender groups was applied to select controls (n = 833) for the remaining 833 patients on whom genotyping was carried out for the SNPs identified at the first stage. The 1,008 normal controls consisted of 314 individuals from the Taiwan Han Chinese Cell and Genome Bank [16], 551 individuals from the Cardiovascular Disease Risk Factor Two-Township Study [17] and 143 individuals from the Nutrition and Health Survey in Taiwan, 2005–2008 [18]. The male-female ratio was 2.08 for both the case and control groups. Mean age (standard deviation) was 42.4 (6.2) for female cases, 40.2 (7.7) for male cases, 42.8 (6.7) for female controls and 40.9 (8.3) for male controls.

### Power calculation

Under certain given scenarios as described below, we calculated power of our two-stage case-control association study by GaTS software [19]. Given an additive-effect disease model with a prevalence of 13.4% for YOH [18], a genetic relative risk of 2, and a disease allele frequency of 0.2–0.4, the power of our two-stage analysis was 0.87–0.90 for a stringent test size of 5.45×10^−7^. The power was reduced to 0.29–0.38, if the genetic relative risk was reduced to 1.5. If the disease followed a multiplicative-effect model with a disease prevalence of 13.4%, a genetic relative risk of 2, and a disease allele frequency of 0.2–0.4, the power of our two-stage analysis increased to 0.96–0.99 for a stringent test size of 5.45×10^−7^. The power was reduced to 0.38–0.56, if the genetic relative risk was reduced to 1.5.

### Inclusion criteria and auxiliary measurements

Inclusion criteria for YOH patients were defined as follows: (1) a systolic blood pressure (SBP)≥140 mmHg and/or diastolic blood pressure (DBP)≥90 mmHg over a 2-month period or, for those who were on anti-hypertensive medication, SBP/DBP≥120/80 mmHg at two consecutive visits over a 2-month period; (2) an initial diagnosis of hypertension between 20 and 51 years of age; (3) no secondary causes of hypertension (such as chronic renal disease, renal arterial stenosis, primary aldosteronism, coarctation of the aorta, thyroid disorders, Cushing's syndrome and pheochromocytoma), which were ruled out through extensive clinical investigations (including blood chemistry, renal function tests, endocrine procedures and abdominal sonogram); (4) a fasting glucose level <126 mg/dl and no previous diagnosis of diabetes mellitus; (5) a body mass index <35 kg/m^2^; (6) having both sides of parents and grandparents identifying themselves as Han Chinese; (7) being a legal resident of Taiwan.

Standard protocols for blood pressure measurements established by the Nutrition and Health Survey in Taiwan [18] were followed by all above studies. Blood pressure was measured three times with two consecutive pulse measurements in between using the Omega 1400 NBP (Invivo Research Laboratories Inc., Orlando, FL, USA). The average of the last two blood pressure measurements was used to confirm the hypertension status. In addition, personal interviews administered by trained nurses ascertained information on socio-demographics, lifestyle and personal habits (smoking, drinking and physical activity) and medical history and medications. For each eligible subject, 17.5 ml of venous blood from an antecubital vein was drawn into a Vacutainer(R) tube (BD, Franklin Lakes, NJ, USA) for clinical chemistry, and 5 ml was drawn into a sodium citrate−containing Monovette tube (Sarstedt AG & Co., Postfach, Nümbrecht, Germany) for DNA extraction.

### SNP genotyping

In the first-stage association mapping, GWAS, YOH cases (n = 175) and normotensive controls (n = 175) were genotyped with the Affymetrix Human Mapping 100K Set (Affymetrix, San Diego, CA, USA), which contains 116,204 SNPs with a median inter-marker distance of 8.5 kb and 92% genome coverage within 100 kb of a SNP. Genomic DNA was isolated from leukocytes using a Puregene kit (Gentra Systems, Minneapolis, MN, USA) for genomic DNA isolation. The DNA concentration was quantified and adjusted to 50 ng/µl using a NanoDrop ND-1000 Spectrophotometer (NanoDrop Technologies, DE, USA). Genotyping of each individual was performed with 500 ng genomic DNA according to the GeneChip Mapping Assay Protocol and the BRLMM (Bayesian Robust Linear Model with Mahalanobis distance classifier algorithm) was used to call genotype data.

In the second-stage association mapping, CMAS, the SNPs identified in GWAS were genotyped with Sequenom MassArray (Sequenom, San Diego, CA, USA) for 833 YOH patients and 833 normotensive controls. The DNA concentration of each individual was measured fluorometrically and then diluted to 25 ng/µl using the PicoGreen dsDNA quantification reagent (Molecular Probes, Eugene, OR, USA). PCR primers and primer extension probes were designed using SpectroDESIGNER software (Sequenom), and all PCR amplifications and primer extension reactions were generated by PCR-ABI 9700 thermocyclers (Applied Biosystems, Foster City, CA, USA). PCR products were transferred from the microplate to a 384-well MassARRAY using SpectroCHIP (Sequenom). The mass spectrum from time-resolved spectra was analyzed and recorded using a MassARRAY mass spectrometer (Sequenom), and each spectrum was then quantified and called using SpectroTYPER and SpectroREADER software (Sequenom), respectively.

### Statistical methods

This study conducted a two-stage association study in humans consisting of GWAS at the first stage and CMAS at the second stage. The detailed procedures are described as follows.

First, we evaluated SNP/genotyping quality by examining the genotyping call rate (GCR), the status of Hardy-Weinberg Equilibrium (HWE) and the minor allele frequency (MAF). The minimum GCR for 350 samples was 0.972. Using the ALLELE procedure of SAS software (SAS Institute, Inc. Cary, NC, USA), we examined HWE using the Markov-Chain Monte-Carlo exact HWE test [20] with one million permutations. Among 112,990 autosomal SNPs, 410 deviated from HWE with a −log_10_(pFDR)>3 (pFDR is defined in Procedure 6); these were excluded from further analysis. Then, 838 SNPs with a GCR<0.9 were excluded. Finally, 20,029 SNPs with a MAF<0.01 were also removed. The remaining 91,713 SNPs were used for further GWAS analysis.

Second, we evaluated population admixture of the Taiwanese population by using STRUCTURE software [21] and genomic control analyses [22]. For the former analysis, we considered the number of populations was K = 3 (Minna, Hakka and Mainlander) under an admixture model. Admixture proportions of all samples in normotensive group and hypertensive group were calculated respectively using STRUCTURE software [21]. For the latter analysis, variance inflation fraction, max{1, square(median of trend test statistics)/square(0.675)}, was calculated by the CASECONTROL procedure of SAS software (SAS Institute, Inc.).

Third, we performed genome-wide single-locus association tests using exact conditional logistic regressions [23], [24], where a dichotomous disease status of YOH was regressed on SNP genotypes in either a nominal genotype coding system (i.e., *AA*, *AB* and *BB*) or an ordinal genotype coding system (i.e., 0, 1 and 2 of allele *A*). Genetic effects of SNPs were examined using one million Monte Carlo samples generated from a hybrid network and Monte Carlo algorithm [25], [26] by the LOGISTIC procedure of SAS software (SAS Institute, Inc.). Throughout this paper, we use the term CLR-NOMINAL analysis to describe the procedure of fitting a conditional logistic regression model to associate hypertension with a nominal-genotype-coding variable; the term CLR-ORDINAL analysis describes fitting the same model with an ordinal-genotype-coding variable.

Fourth, we performed genome-wide multilocus association tests using either the haplotype association test or p-value combination test. The genome-wide haplotype association tests combined haplotype trend regression [27] and a sliding-window procedure to scan the human genome chromosome by chromosome. Multiple moving window sizes of 3, 5, 7 and 9 SNPs were used. Haplotype frequencies were estimated using the composite haplotype method [28], which requires less computational time than the expectation-maximization algorithm. Haplotypes with low frequencies were excluded using three thresholds of minimum haplotype frequencies, 0.01, 0.05 or 0.10. The analysis was carried out using HelixTree software (Golden Helix, Inc. Bozeman, MT, USA).

The genome-wide p-value combination test combined a truncated product p-value procedure [29] and a sliding-window procedure to scan the human genome chromosome by chromosome, where the p values were those obtained from the previous genome-wide single-locus association tests (CLR-NOMINAL or CLR-ORDINAL). Multiple moving window sizes of 3, 5, 7 and 9 were applied. The analysis was carried out using the PSMOOTH procedure of SAS software (SAS Institute, Inc.).

Fifth, we performed genome-wide pair-wise SNP-SNP interaction tests for all possible combinations by testing whether the odds ratios for the combined genotypes significantly differed between case and control groups. PLINK software [30] was used. SNP pairs identified were further verified by exact conditional logistic regression models with interactive covariate(s) based on one million Monte Carlo samples, where both nominal and ordinal genotyping coding systems were considered. Significance of an interactive effect with four degrees of freedom for a nominal genotyping coding system and an interactive effect with one degree of freedom for an ordinal genotyping coding system were examined by a type III analysis, respectively. The conditional logistic regression analysis was run using the LOGISTIC procedure of SAS software (SAS Institute, Inc.).

Sixth, we performed multiple testing corrections. Multiplicity of testing was adjusted using either the false discovery rate (FDR) [31], pFDR, or a stringent p-value threshold in various stages of analyses. SNPs with −log_10_(pFDR)>3 in HWE tests were excluded from the subsequent analysis. SNPs, haplotype sets and SNP triplets with −log_10_(pFDR)>3 were considered significant for marker-trait associations. SNP pairs with a −log_10_(p)>8 for interaction were identified as significant interactive pairs. All of the SNP markers identified by any of the GWAS procedures were annotated using GENOWATCH software [32]. For those SNPs with at least one gene located within 100 kb of the flanking regions, the SNP-hypertension associations were further examined with more samples in the CMAS.

CMAS was carried out with 1,008 YOH patients and 1,008 normotensive controls. We used two analysis strategies, independent data analysis and joint data analysis. The fomer strategy was to analyze only the independent samples in CMAS (i.e., 833 YOH patients and 833 controls) and the later strategy was to analyze the combined samples in GWAS and CMAS (i.e., 1,008 YOH patients and 1,008 normotensive controls). Age and gender were adjusted in the analyses. Genotyping quality control procedures were identical to those used during the first stage. An unconditional logistic regression model with either a nominal-genotype-coding covariate (ULR-NOMINAL analysis) or an ordinal-genotype-coding covariate (ULR-ORDINAL analysis) was carried out. Association/interaction tests were performed to confirm the previous findings in the GWAS. Odds ratios and the corresponding 95% confidence intervals were calculated to estimate the effect sizes of the identified SNPs. In addition, the linkage disequilibria (LD) structure of the identified contiguous SNPs was examined using the HAPLOVIEW software [33]. Haplotype-trait association was examined based on a likelihood ratio test [34]. Ten thousand permutations were performed to calculate empirical p values of overall tests and individual haplotype tests.

## Results

### GWAS at the first stage

Using 91,713 SNPs with good quality (see the discussion of statistical methods), we investigated marginal effects (genome-wide single-locus association test), joint effects (genome-wide multilocus association test) and interactive effects (genome-wide pair-wise interaction test) of SNPs on YOH.

First, STRUCTURE software and genomic control analyses were performed to evaluate population admixture/stratification. The results from STRUCTURE shows that the overall admixture structures in our case samples and control samples are very similar, suggesting the admixture in our population should not cause spurious association in our association study. In addition, the genomic control analysis shows that the variance inflation fraction was 1.097, close to 1, also suggesting the impact of population admixture/stratification on our association study is not significant. The conclusion is similar to the findings in the previous studies [35]–[37].

Second, genome-wide single-locus association tests were carried out to detect marginal genetic effects of YOH. Exact p values were calculated for the CLR-NOMINAL and CLR-ORDINAL analyses to associate YOH with SNPs. After applying an FDR correction to the p values, no SNPs satisfied −log_10_(pFDR)>3 (see **Figures S1(A) and S1(B)**). That is, no individual SNP was significantly associated with the status of YOH in this study.

Third, we further examined the effects of multiple SNPs on YOH by two types of genome-wide multilocus association analyses: p-value combination analysis and haplotype analysis with sliding windows of 3, 5, 7 and 9 SNPs. Because the analyses of different window sizes identified similar association regions, here we show only the results of window size 3.

**Figure 1:**
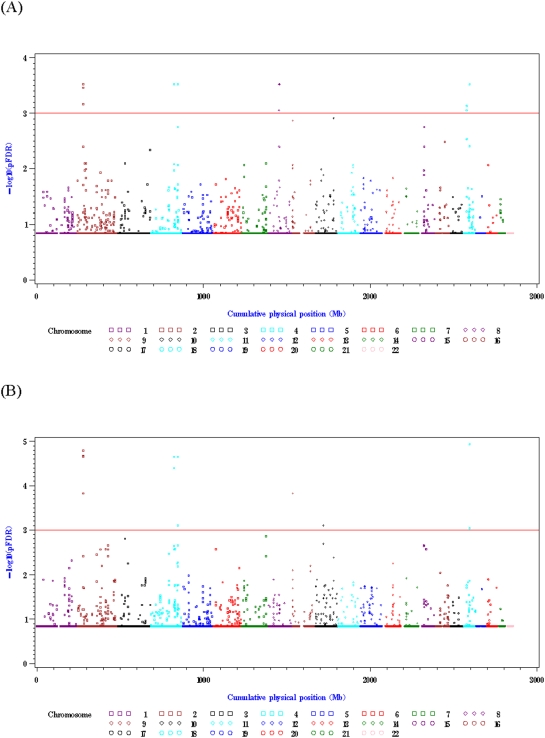
Results of genome-wide p-value combination analysis using p values from CLR-NOMINAL or CLR-ORDINAL analyses. The *y* axis denotes −log_10_(pFDR), and the *x* axis denotes cumulative physical positions on autosomes. The red reference line signifies −log_10_(pFDR) = 3. (A) Results of p-value combination tests based on p-values from a CLR-NOMINAL analysis; (B) Results of p-value combination tests based on p-values from a CLR-ORDINAL analysis.

The p-value combination analysis integrated p values from either the CLR-NOMINAL or CLR-ORDINAL analyses. The analyses identified 20 significant triplets of SNPs with −log_10_(pFDR)>3 (see **Figures 1(A) and 1(B)**). Numerical results and gene information of anchor (central) markers of the 20 identified SNP triplets are summarized in **Table 1**. Among them, 13 triplets were identified by one analysis and seven by two analyses. Among the seven triplets identified by the two analyses, we focused on the three triplets located in known or hypothetical gene regions (bold in **Table 1**). Note that the unadjusted p values (in −log_10_ scale) of the three triplets were greater than 7. The first triplet was rs9308945-rs6711736-rs6729869 on chromosome 2; the second triplet was rs6711736-rs6729869-rs10495809 on chromosome 2; the third triplet was rs10517739-rs1444280-rs10517740 on chromosome 4. The two triplets on chromosome 2 contained two overlapping SNPs, forming a SNP quartet. Seven distinct SNPs in the three SNP triplets were genotyped for more samples with the Sequenom's MassARRAY and further analyzed statistically in a CMAS, which is described below. Genome-wide haplotype trend regression was also carried out but did not identify any windows (i.e., triplets of SNPs) with −log_10_(pFDR)>3 (see **Figures S2(A)**, **S2(B) and S2(C)**).

**Table 1:** A list of anchor SNPs identified by the p-value combination analysis in GWAS.

CHR	RS	PP	GI	CLR-NOMINAL	CLR-NOMINAL	CLR-ORDINAL	CLR-ORDINAL
				−log_10_(p)	−log_10_(pFDR)	−log_10_(p)	−log_10_(pFDR)
2	**rs6711736**	**34196635**	**Hypothetical**	**7.48527**	**3.52284**	**9.16600**	**4.68070**
	**rs6729869**	**34196845**	**Hypothetical**	**7.04924**	**3.16599**	**9.02237**	**4.66200**
	rs10495809	34216890	Intergenic	7.38445	3.46342	9.46051	4.79912
	rs1346007	34217210	Intergenic	5.79457	2.39848	7.84643	3.82731
4	rs6854244	138479489	Intergenic	7.76575	3.52284	8.84525	4.65691
	rs10519412	138487534	Intergenic	7.79596	3.52284	8.51803	4.40070
	rs10517739	162515975	Intergenic	6.43381	2.75013	7.02313	3.10209
	**rs1444280**	162516134	***FSTL5***	**7.88286**	**3.52284**	**8.84119**	**4.65691**
8	rs4737649	64515583	*IFITM8P*	7.79134	3.52284	4.31484	1.54609
	rs1431587	64516073	*IFITM8P*	7.70481	3.52284	3.80069	1.31314
	rs1367807	64544666	Intergenic	6.83890	3.05256	3.44477	1.13168
	rs831738	70313702	Intergenic	7.49105	3.52284	3.65335	1.23307
	rs705994	70313758	Intergenic	7.50762	3.52284	3.61202	1.21483
	rs705993	70314229	Intergenic	7.53247	3.52284	3.62344	1.21852
9	rs945658	1606639	Intergenic	6.59932	2.86734	3.9664	3.82731
10	rs2620887	49730508	*WDFY4*	5.15365	1.88658	7.04094	3.10209
18	rs10513909	8326569	Intergenic	6.98389	3.13540	4.76805	1.76466
	rs10513910	18326428	Intergenic	6.85253	3.05256	3.96647	1.38965
	rs9284423	36256475	Intergenic	8.48521	3.52284	9.90126	4.93883
	rs9304221	36256659	Intergenic	6.02669	2.40668	6.92917	3.04592

The p-value combination analysis with a window size of 3 identified 20 triplets of SNPs in GWAS. Chromosome (CHR), RS number (RS), physical position (PP) and gene information (GI) of the anchor (central) marker of each SNP triplet are shown. The last four columns represent unadjusted p value (−log_10_(p)) and adjusted p value (−log_10_(pFDR)) of the p-value combination analysis based on single-locus p values from a CLR-NOMINAL analysis [model: logit{Prob(Y = YOH|I_11_,I_12_)} = a_0_+a_11_×I_11_+a_12_×I_12_] and a CLR-ORDINAL analysis [model: logit{Prob(Y = YOH|X)} = a_0_+a_1_×X], where Y is YOH status, I_1j_ is an indicator of the three genotypes (j = 1, 2) of a SNP and X is a variable for the number of reference alleles. Anchor markers that had −log_10_(pFDR)>3 for both tests and were located in known or hypothetical gene regions are highlighted in bold font.

Fourth, the interactive effects of all possible pairs of SNPs (more than 4.2 billion SNP pairs) were exhaustively examined. Numerical results and gene information of the identified top 10 SNP pairs are summarized in **Table 2**. The 10 SNP pairs satisfied the following two conditions: (1) the difference test of odds ratios between case and control groups for the combined genotypes showed −log_10_(p)>8 (see column “ORT” in **Table 2**) and (2) at least one SNP from the pair was located in a gene region. Except for SNP pair rs1526555-rs765899, the significance of the remaining 9 SNP pairs was also confirmed using interaction tests of a CLR-NOMINAL analysis (see column “Nominal” in **Table 2**) and a CLR-ORDINAL analysis (see column “Ordinal” in **Table 2**) based on the same data, where significance of the CLR-NOMINAL and CLR-ORDINAL analyses were evaluated by considering an FDR correction of 10 replication tests. The significance of the 10 SNP pairs was also re-examined in a CMAS, the results of which are described below.

**Table 2:** A list of SNP pairs identified by SNP-SNP interaction tests in GWAS.

First SNP in an interactive pair						*Second SNP in an interactive pair*						*Interaction tests* [−log_10_(p)]		
CHR	RS	PP	GI	GCR	MAF	CHR	RS	PP	GI	GCR	MAF	ORT	Nominal	Ordinal
7	rs1526555	11579529	*KIAA0960*	99.4	0.140	14	rs765899	68033499	*RAD51L1*	92.0	0.326	8.9073	2.9786	5.0546
11	rs7950640	58620981	*LOC643652*	100	0.387	7	rs2214310	25152103	*xC7orf9*	99.7	0.454	8.7347	3.9040	5.7904
12	rs10506451	61520532	*PPM1H*	100	0.357	6	rs1115620	113578211	*LOC643884*	99.4	0.496	8.5318	4.2654	5.8117
11	rs7950640	58620981	*LOC643652*	100	0.387	7	rs994531	25152938	*xC7orf9*	99.4	0.441	8.3949	3.7367	5.6075
6	rs1886985	76773462	*IMPG1*	100	0.491	20	rs2206416	39994257	Intergenic	100	0.486	8.3879	4.7945	6.7676
11	rs10488767	109964045	*ARHGAP20*	100	0.317	16	rs10500328	5747879	Intergenic	100	0.404	8.2666	3.6095	5.6956
12	rs10506451	61520532	*PPM1H*	100	0.357	6	rs2502397	113569072	*LOC643884*	100	0.499	8.1975	4.0066	5.6377
1	rs618171	215537693	*LOC643717*	100	0.443	7	rs7805441	77766109	*MAGI2*	100	0.451	8.1824	3.5028	5.3108
6	rs1886985	76773462	*IMPG1*	100	0.491	20	rs6129969	39967987	Intergenic	99.1	0.419	8.1167	3.7163	6.1904
7	rs1526555	11579529	*KIAA0960*	99.4	0.140	14	rs2331706	68031318	*RAD51L1*	100	0.364	8.0217	3.1281	4.9362

Interactive SNP pairs are listed in their order of significance. For each SNP of an interactive pair, chromosome (CHR), RS number (RS), physical position (PP), gene information (GI), genotyping call rate (GCR(%)) and minor allele frequency (MAF) are shown. Finally, the −log_10_(p) value from three interaction tests is shown: (1) the difference test of odds ratio (ORT), (2) an interaction test of the CLR-NOMINAL analysis [model: logit{Prob(Y = YOH|I_11_,I_12_,I_21_,I_22_,I_11_×I_21_,I_11_×I_22_,I_12_×I_21_,I_12_×I_22_)} = a_0_+a_11_×I_11_+a_12_×I_12_+a_21_×I_21_+a_22_×I_22_+b_1_×I_11_×I_21_+b_2_×I_11_×I_22_+b_3_×I_12_×I_21_+b_4_×I_12_×I_22_] and (3) an interaction test of the CLR-ORDINAL analysis [model: logit{Prob(Y = YOH|X_1_, X_2_, X_1_×X_2_)} = a_0_+a_1_×X_1_+a_2_×X_2_+a_3_×X_1_×X_2_], where Y is YOH status, I_ij_ is an indicator of the three genotypes (j = 1, 2) of SNP i (i = 1, 2) and X_i_ denotes the number of reference alleles of SNP i (i = 1, 2).

In summary, the GWAS identified three SNP triplets with p-value combination tests and 10 pairs of SNPs with significant interactive effects that are located in gene regions. The three triplets contain seven distinct SNPs and the 10 interactive pairs contain 16 distinct SNPs. All of the resulting 23 SNPs were genotyped in the CMAS.

### CMAS at the second stage

For confirmatory purposes, the 23 SNPs were genotyped for 833 YOH patients and 833 normal controls. Summary statistics and p values of single-locus association tests based on the independent samples (833 YOH patients and 833 normal controls) and on the combined samples (1,008 YOH patients and 1,008 normal controls) are presented (see **Table 3**). Odds ratios and 95% confidence intervals were calculated based on either the independent samples or the combined samples (see **Table 4**). After considering a multiple testing correction (−log_10_(pFDR)>3), no significant results were found for single-locus association tests. This result suggests that a single SNP may not be capable of producing a detectable YOH-SNP association. This conclusion is consistent with our GWAS findings.

**Table 3:** A list of SNPs initially identified by GWAS and re-examined in CMAS.

M	CHR	RS	NT	GI	*Combined samples*					*Independent samples*				
					GCR	MA:MAF	HWE	ULR-NOMINAL	ULR-ORDINAL	GCR	MA:MAF	HWE	ULR-NOMINAL	ULR-ORDINAL
P	2	rs9308945	A/G	Hypothetical	99.8	A: 0.393	1.0000	0.0007	0.0002	99.8	A: 0.394	0.6162	0.0539	0.0215
P	2	rs6711736	A/G	Hypothetical	97.3	G: 0.392	0.3921	0.0004	0.0001	96.8	G: 0.393	0.1690	0.0226	0.0130
P	2	rs6729869	A/T	Hypothetical	99.7	T: 0.363	0.5964	0.0017	0.0011	99.6	T: 0.362	0.2330	0.0345	0.0589
P	2	rs10495809	A/G	Intergenic	99.4	A: 0.453	0.9480	0.0260	0.0076	99.3	A: 0.453	0.5784	0.3715	0.1607
P	4	rs10517739	C/T	Intergenic	99.6	C: 0.316	0.5590	0.6727	0.9571	99.5	C: 0.311	0.3964	0.0489	0.0368
P	4	rs1444280	C/G	*FSTL5*	97.5	C: 0.310	0.3669	0.5331	0.8619	97.8	C: 0.304	0.2256	0.0121	0.0127
P	4	rs10517740	G/T	Intergenic	91.4	T: 0.210	<0.0001	<0.0001	<0.0001	89.6	T: 0.200	<0.0001	<0.0001	<0.0001
I	7	rs1526555	A/C	*KIAA0960*	95.9	A: 0.130	0.0101	0.3549	0.1685	95.2	A: 0.128	0.0921	0.7118	0.4393
I	14	rs2331706	C/T	*RAD51L1*	99.9	T: 0.377	0.8912	0.4841	0.2285	99.9	T: 0.380	1.0000	0.3090	0.1298
I	14	rs765899	C/T	*RAD51L1*	98.7	T: 0.377	0.6309	0.4011	0.1873	99.5	T: 0.378	1.0000	0.3330	0.1383
I	11	rs7950640	C/G	*LOC643652*	99.7	C: 0.363	0.7408	0.9408	0.8137	99.6	C: 0.357	0.8203	0.7638	0.5403
I	7	rs2214310	C/T	*xC7orf9*	99.7	C: 0.482	0.6158	0.7633	0.6486	99.7	C: 0.488	0.8881	0.8882	0.9074
I	7	rs994531	A/G	*xC7orf9*	99.6	A: 0.478	0.4090	0.8472	0.6270	99.6	A: 0.485	0.8385	0.9398	0.7262
I	12	rs10506451	A/G	*PPM1H*	99.7	A: 0.362	0.8935	0.1134	0.0418	99.6	A: 0.363	0.3631	0.1468	0.0719
I	6	rs1115620	A/T	*LOC643884*	98.0	T: 0.482	0.5292	0.8051	0.5189	97.7	T: 0.477	0.5228	0.8794	0.8378
I	6	rs2502397	A/G	*LOC7805441*	99.9	A: 0.488	0.6640	0.2685	0.1465	99.9	A: 0.485	0.5768	0.5201	0.2660
I	6	rs1886985	A/G	*IMPG1*	99.9	G: 0.491	0.9494	0.8774	0.9232	99.8	G: 0.488	1.0000	0.9408	0.9257
I	20	rs6129969	C/T	Intergenic	99.9	T: 0.392	0.2834	0.3593	0.3800	99.9	T: 0.385	0.0386	0.0539	0.7652
I	20	rs2206416	C/T	Intergenic	98.7	C: 0.368	<0.0001	0.7193	0.9571	98.4	C: 0.337	<0.0001	0.0545	0.6498
I	11	rs10488767	A/G	*ARGAP20*	98.7	A: 0.285	0.4337	0.7580	0.4661	98.4	A: 0.278	0.9359	0.4576	0.3175
I	16	rs10500328	A/G	Intergenic	87.2	A: 0.158	0.0001	0.3558	0.2359	84.6	A: 0.096	0.0268	0.0407	0.0417
I	1	rs618171	A/G	*LOC643717*	99.5	A: 0.464	0.2786	0.2278	0.1167	99.3	A: 0.468	0.3009	0.4699	0.3065
I	7	rs7805441	C/T	*MAGI2*	99.7	T: 0.467	0.2573	0.1722	0.1993	99.6	T: 0.471	0.1860	0.3112	0.5076

For each SNP, the method used (M), chromosome (CHR), RS number (RS), nucleotide types (NT) and gene information (GI) are shown. The results for combined samples and independent samples are shown as follows: (1) genotyping call rate (GCR(%)), (2) minor allele and minor allele frequency (MA:MAF), (3) p values of the exact HWE test (HWE), (4) exact p value of the ULR-NOMINAL analysis [model: logit{Prob(Y = YOH|I_11_,I_12_,I_Gender_,Z_Age_} = a_0_+a_11_×I_11_+a_12_×I_12_+a_2_×I_Gender_+a_3_×Z_Age_] and (5) exact p value of the ULR-ORDINAL analysis [model: logit{Prob(Y = YOH|X,I_Gender_,Z_Age_)} = a_0_+a_1_×X+a_2_×I_Gender_+a_3_×Z_Age_], where Y is YOH status, I_1j_ is an indicator of the three genotypes (j = 1, 2) of a SNP, I_Gender_ is an indicator of gender, Z_Age_ is a covariate for age, and X is a variable for the number of reference alleles.

**Table 4:** Odds ratios and 95% confidence intervals of SNPs identified by a p-value combination method and a SNP-SNP interaction analysis in GWAS.

M	CHR	RS	NT	GI	*Combined samples*			*Independent samples*		
					OR_1_	OR_2_	OR_3_	OR_1_	OR_2_	OR_3_
P	2	rs9308945	A/G	Hypothetical	0.59 (0.45, 0.78)	0.84 (0.69, 1.02)	0.79 (0.69, 0.89)	1.07 (0.80, 1.44)	1.33 (1.00, 1.77)	0.85 (0.74, 0.98)
P	2	rs6711736	A/G	Hypothetical	1.72 (1.31, 2.26)	1.42 (1.10, 1.85)	1.29 (1.13, 1.47)	1.51 (1.12, 2.02)	1.39 (1.05, 1.85)	1.20 (1.04, 1.38)
P	2	rs6729869	A/T	Hypothetical	1.68 (1.26, 2.23)	1.50 (1.13, 1.98)	1.24 (1.09, 1.42)	1.46 (1.08, 1.99)	1.47 (1.08, 1.99)	1.15 (1.00, 1.32)
P	2	rs10495809	A/G	Intergenic	1.40 (1.09, 1.80)	1.23 (1.00, 1.50)	1.19 (1.05, 1.34)	1.21 (0.92, 1.60)	1.11 (0.89, 1.39)	1.10 (0.96, 1.26)
P	4	rs10517739	C/T	Intergenic	0.93 (0.68, 1.26)	1.06 (0.88, 1.27)	1.00 (0.87, 1.14)	1.22 (0.87, 1.72)	1.28 (1.05, 1.57)	1.17 (1.01, 1.36)
P	4	rs1444280	C/G	*FSTL5*	0.94 (0.69, 1.28)	1.09 (0.90, 1.31)	1.01 (0.88, 1.16)	1.26 (0.89, 1.79)	1.36 (1.11, 1.67)	1.21 (1.04, 1.41)
P	4	rs10517740	G/T	Intergenic	0.52 (0.37, 0.74)	2.59 (1.74, 3.86)	0.44 (0.37, 0.52)	0.53 (0.37, 0.78)	5.27 (3.24, 8.56)	0.39 (0.32, 0.47)
I	7	rs1526555	A/C	*KIAA0960*	1.18 (0.67, 2.06)	1.17 (0.94, 1.46)	1.14 (0.95, 1.36)	1.26 (0.67, 2.39)	1.06 (0.83, 1.36)	1.08 (0.89, 1.33)
I	14	rs2331706	C/T	*RAD51L1*	0.85 (0.65, 1.12)	0.92 (0.71, 1.20)	0.93 (0.81, 1.05)	0.80 (0.59, 1.07)	0.87 (0.65, 1.16)	0.90 (0.78, 1.03)
I	14	rs765899	C/T	*RAD51L1*	0.85 (0.65, 1.12)	0.95 (0.73, 1.24)	0.92 (0.81, 1.04)	0.81 (0.60, 1.09)	0.89 (0.67, 1.20)	0.90 (0.78, 1.04)
I	11	rs7950640	C/G	*LOC643652*	1.02 (0.77, 1.34)	1.03 (0.86, 1.25)	1.02 (0.89, 1.16)	1.07 (0.78, 1.46)	1.08 (0.88, 1.33)	1.05 (0.91, 1.21)
I	7	rs2214310	C/T	*xC7orf9*	1.06 (0.82, 1.35)	1.08 (0.88, 1.33)	1.03 (0.91, 1.17)	1.01 (0.77, 1.33)	1.06 (0.84, 1.33)	1.01 (0.88, 1.16)
I	7	rs994531	A/G	*xC7orf9*	1.06 (0.83, 1.36)	1.06 (0.86, 1.30)	1.03 (0.91, 1.17)	1.05 (0.80, 1.38)	1.02 (0.81, 1.29)	1.03 (0.90, 1.17)
I	12	rs10506451	A/G	*PPM1H*	1.34 (1.01, 1.77)	1.11 (0.92, 1.34)	1.14 (1.01, 1.30)	1.36 (1.00, 1.86)	1.07 (0.87, 1.32)	1.14 (0.99, 1.32)
I	6	rs1115620	A/T	*LOC643884*	0.92 (0.71, 1.18)	0.95 (0.76, 1.18)	0.96 (0.85, 1.09)	0.98 (0.74, 1.29)	1.03 (0.81, 1.33)	0.99 (0.86, 1.13)
I	6	rs2502397	A/G	*LOC7805441*	1.21 (0.94, 1.54)	1.03 (0.83, 1.27)	1.10 (0.97, 1.24)	1.17 (0.89, 1.54)	1.05 (0.84, 1.33)	1.08 (0.94, 1.24)
I	6	rs1886985	A/G	*IMPG1*	1.01 (0.79, 1.29)	0.96 (0.77, 1.19)	1.01 (0.89, 1.14)	0.99 (0.75, 1.29)	0.96 (0.76, 1.22)	0.99 (0.87, 1.14)
I	20	rs6129969	C/T	Intergenic	0.93 (0.72, 1.22)	1.07 (0.83, 1.39)	0.95 (0.83, 1.07)	1.07 (0.80, 1.44)	1.33 (1.00, 1.77)	0.98 (0.85, 1.13)
I	20	rs2206416	C/T	Intergenic	0.98 (0.79, 1.22)	1.08 (0.87, 1.33)	1.00 (0.90, 1.11)	0.89 (0.70, 1.14)	1.27 (0.99, 1.63)	0.97 (0.87, 1.09)
I	11	rs10488767	A/G	*ARGAP20*	1.12 (0.81, 1.55)	1.04 (0.87, 1.25)	1.05 (0.92, 1.21)	1.26 (0.88, 1.82)	1.02 (0.84, 1.26)	1.08 (0.93, 1.26)
I	16	rs10500328	A/G	Intergenic	0.92 (0.59, 1.41)	0.85 (0.67, 1.07)	0.90 (0.76, 1.07)	0.95 (0.45, 1.98)	0.68 (0.51, 0.92)	0.78 (0.62, 0.99)
I	1	rs618171	A/G	*LOC643717*	0.81 (0.64, 1.04)	0.96 (0.78, 1.18)	0.91 (0.80, 1.03)	0.86 (0.66, 1.13)	0.99 (0.79, 1.25)	0.93 (0.81, 1.07)
I	7	rs7805441	C/T	*MAGI2*	1.16 (0.90, 1.49)	0.95 (0.76, 1.19)	1.09 (0.96, 1.23)	1.08 (0.82, 1.42)	0.91 (0.71, 1.16)	1.05 (0.91, 1.20)

For each SNP, the method used (M), chromosome (CHR), RS number (RS), nucleotide types (NT) and gene information (GI) are shown. Two genotypic odds, OR_1_ and OR_2_, from the ULR-NOMINAL analysis [model: logit{Prob(Y = YOH|I_11_,I_12_,I_Gender_,Z_Age_)} = a_0_+a_11_×I_11_+a_12_×I_12_+a_2_×I_Gender_+a_3_×Z_Age_] and one allelic odds ratio, OR_3_, from the ULR-ORDINAL analysis [model: logit{Prob(Y = YOH|X,I_Gender_,Z_Age_)} = a_0_+a_1_×X+a_2_×I_Gender_+a_3_×Z_Age_] were calculated, where Y is YOH status, I_1j_ is an indicator of the three genotypes (j = 1, 2) of a SNP, I_Gender_ is an indicator of gender, Z_Age_ is a covariate for age, and X is a variable for the number of reference alleles. The results for combined samples and independent samples are shown.

Next, we verified the significant findings obtained from genome-wide multilocus association tests and the interaction tests. We included only SNPs that passed the quality criteria in the CMAS. Among the 23 SNPs, two SNPs (rs10517740 and rs10500328) had a GCR<0.9, and three SNPs (rs10517740, rs2206416 and rs10500328) significantly deviated from HWE (see **Table 3**); these were excluded from the subsequent analysis. In addition, one SNP triplet (rs104517739, rs1444280 and rs10517740) on chromosome 4 contained the poor quality SNP rs10517740, resulting in the exclusion of two SNPs (rs104517739 and rs1444280). On the other hand, an interactive SNP pair (rs10488767-rs10505328) contained one of the three poor-quality SNPs, resulting in the exclusion of a SNP (rs10488767). Therefore, we examined only four distinct SNPs (rs9308945, rs6711736, rs6729869 and rs10495809) for p-value combination and examined 13 SNPs (rs618171, rs7805441, rs1115620, rs10506451, rs2502397, rs1886985, rs6129969, rs2214310, rs7950640, rs994531, rs1526555, rs2331706 and rs765899) for genetic interaction. We carried out confirmation analyses on the following three SNP groups: (1) four distinct SNPs resulting from the two SNP triplets on chromosome 2, and (2) 13 distinct SNPs resulting from 8 interactive SNP pairs. All results are summarized in **Table 5** and **Table 6**.

**Table 5:** Confirmatory p-value combination analysis.

CHR	SNP pair/triplet/quartet	*Combined samples*		*Independent samples*	
		ULR-NOMINAL	ULR-ORDINAL	ULR-NOMINAL	ULR-ORDINAL
		−log_10_(p)	−log_10_(p)	−log_10_(p)	−log_10_(p)
2	rs9308945 - rs6711736	5.4738	6.5331	1.3426	2.8468
2	rs6711736 - rs6729869	5.1218	5.8471	2.4989	1.5654
2	rs6729869 - rs10495809	3.5135	4.1433	1.1672	1.0110
2	rs9308945 - rs6711736 - rs6729869	7.1739	8.3979	2.1520	2.4655
2	rs6711736 - rs6729869 - rs10495809	5.8055	6.9586	2.1520	1.3721
2	rs9308945 - rs6711736 - rs6729869- rs10495809	7.8469	9.5155	1.9102	2.2028

SNP pairs, triplets and quartets that were identified by the p-value combination method in GWAS were verified in CMAS. Truncated product p-value statistics were calculated by combing single p values from the previous single-locus ULR-NOMINAL analysis or ULR-ORDINAL analysis in CMAS. The exact p values (in −log_10_ scale) for the combined and independent samples are shown.

**Table 6:** Confirmatory interaction analysis.

*The first SNP in an interactive pair*				*The second SNP in an interactive pair*				*Combined samples*		*Independent samples*	
CHR	RS	NT	GI	CHR	RS	NT	GI	ULR-NOMINAL	ULR-ORDINAL	ULR-NOMINAL	ULR-ORDINAL
								−log_10_(p)	−log_10_(p)	−log_10_(p)	−log_10_(p)
1	rs618171	A/G	*LOC643717*	7	rs7805441	C/T	*MAGI2*	1.2890	1.9355	0.1516	0.0295
6	rs1115620	A/T	*LOC643884*	12	rs10506451	A/G	*PPM1H*	0.8380	1.8210	0.0427	0.0225
6	rs2502397	A/G	*LOC7805441*	12	rs10506451	A/G	*PPM1H*	0.6946	1.5200	0.1375	0.1086
6	rs1886985	A/G	*IMPG1*	20	rs6129969	C/T	Intergenic	4.0000	2.3318	1.2248	0.5629
7	rs2214310	C/T	*xC7orf9*	11	rs7950640	C/G	*LOC643652*	0.3310	0.7582	0.2480	0.7878
7	rs994531	A/G	*xC7orf9*	11	rs7950640	C/G	*LOC643652*	0.4148	0.8589	0.2426	0.6214
7	rs1526555	A/C	*KIAA0960*	14	rs2331706	C/T	*RAD51L1*	1.4622	2.1612	0.1350	0.0956
7	rs1526555	A/C	*KIAA0960*	14	rs765899	C/T	*RAD51L1*	1.3439	2.0410	0.1430	0.0753

For each SNP pair, chromosome (CHR), RS number (RS), nucleotide types (NT) and gene information (GI) are shown. Based on combined samples or independent samples in CMAS, the interactive effect was examined using a ULR-NOMINAL analysis [model: logit{Prob(Y = YOH|I_11_, I_12_,I_21_,I_22_,I_11_×I_21_,I_11_×I_22_,I_12_×I_21_,I_12_×I_22_,I_Gender_,Z_Age_)} = a_0_+a_11_×I_11_+a_12_×I_12_+a_21_×I_21_+a_22_×I_22_+b_1_×I_11_×I_21_+b_2_×I_11_×I_22_+b_3_×I_12_×I_21_+b_4_×I_12_×I_22_+c×I_Gender_+d×Z_Age_] or a ULR-ORDINAL analysis [model: logit{Prob(Y = YOH|X_1_,X_2_,X_1_×X_2_,I_Gender_,Z_Age_))} = a_0_+a_1_×X_1_+a2×X_2_+a_3_×X_1_×X_2_+c×I_Gender_+d×Z_Age_], where Y is YOH status, I_ij_ is an indicator of the three genotypes (j = 1, 2) of SNP i (i = 1, 2), I_Gender_ is an indicator of gender, Z_Age_ is a covariate for age, and X_i_ denotes the number of reference alleles of SNP i (i = 1, 2). The exact p values (in −log_10_ scale) for the combined and independent samples are shown.

First, we confirmed the significance of the four contiguous SNPs (rs9308945, rs6711736, rs6729869 and rs10495809) located in a hypothetical gene on chromosome 2. They were examined by a p-value combination analysis with a window size of 2, 3 and 4 SNPs. The significance for each respective SNP was strengthened after considering the join effect of multiple SNPs (see **Table 5**). The same findings were observed for the independent samples and for the combined samples using either the ULR-NOMINAL or the ULR-ORDINAL analysis. For example, in the analysis of the combined samples, the marginal p values of the ULR-NOMINAL analysis of the four SNPs were 0.0007, 0.0004, 0.0017 and 0.0260, respectively (see **Table 3**). P values were greatly reduced after considering the truncated product p-value method for the SNP pair, triplet and quartet. Results showed that −log_10_(p) values of SNP pairs rs9308945–rs6711736, rs6711736–rs6729869 and rs6729869–rs10495809 were 5.4738, 5.1218 and 3.5135, respectively; −log_10_(p) values of SNP triplets rs9308945–rs6711736–rs6729869 and rs6711736–rs6729869–rs10495809 were 7.1739 and 5.8055, respectively, and −log_10_(p) value of SNP quartet rs9308945–rs6711736–rs6729869–rs10495809 was 7.8469 (see **Table 5**). After applying an FDR correction to the p values, the SNP pairs, triplets and quartet satisfied −log_10_(pFDR)>3.

We further examined this region by considering LD and haplotype analyses. LD structures of the four SNPs in the case-only group, the control-only group and the combined group were highly consistent. The four SNPs formed a strong LD block where coefficients of LD, D′, for any SNP pairs were greater than 0.95 (see **Figures S3(A), **S3(B) and S3(C)****). Haplotype-based association tests for the two SNP triplets rs9308945-rs6711736-rs6729869 and rs6711736-rs6729869-rs10495809 and for the SNP quartet rs9308945-rs6711736-rs6729869-rs10495809 showed that global p values of haplotype-trait association tests were 0.0010, 0.0057 and 0.0055, respectively.

For the SNP triplet rs9308945-rs6711736-rs6729869, two haplotypes presented significantly different distributions between case and control groups. Frequencies of haplotype A-G-T in hypertensive and normotensive groups were 0.3368 and 0.3862, respectively, with a p value of 0.0008 for the difference test. Frequencies of haplotype G-A-A in hypertensive and normotensive groups were 0.6349 and 0.5759, respectively, with a p value of 0.0001. In the SNP triplet rs6711736-rs6729869-rs10495809, two significant haplotypes were identified. Frequencies of haplotype A-A-A in hypertensive and normotensive groups were 0.4694 and 0.4252, respectively, with a p value of 0.0059. Frequencies of haplotype G-T-G in hypertensive and normotensive groups were 0.3328 and 0.3803, respectively, with a p value of 0.0019.

Second, we investigated the 13 SNPs that consisted of 8 significant interactive pairs identified by our GWAS. None of the 13 SNPs showed a significant marginal effect (see **Table 3**). Only the interactive effect of a SNP pair rs1886985-rs6129969 was confirmed in the combined samples (see **Table 6**). SNP pair rs1886985-rs6129969 had −log_10_(p) = 4.0000 for a ULR-NOMINAL analysis and −log_10_(p) = 2.3318 for a ULR-ORDINAL analysis. P-values of the ULR-ORDINAL analyses satisfied −log_10_(pFDR)>3.

## Discussion

Hypertension is a common complex disorder characterized by multifactorial inheritance, polygenic effects and genetic heterogeneity. The complex etiology of hypertension has made it difficult to map disease-related genes. To date, no high-impact genes have been directly linked to the onset of hypertension. In this study, we not only carefully selected the phenotype (i.e., by focusing on YOH) but also employed statistical methods designed to increase the power of our analysis and to overcome genetic complexity. The type of statistical gene mapping method used in gene mapping studies is critical for successfully identifying genes responsible for complex disorders. The single-locus association method, which is useful for the detection of marginal effects, may not be sufficient for the investigation of joint effects and interactive (synergic) effects of complex disorders. To increase the test power, in addition to the single-locus association test, we used various multilocus association methods, including the p-value combination approach [29], haplotype analysis [27], [34] and interaction analysis [23]–[26], [30], to compensate for the limitations of the single-locus association test and to examine fully the genetic complexity of hypertension.

It is a challenge to study genome-wide interactions. In our GWAS, we conducted a two-step genome-wide interaction analysis to examine all possible pair-wise SNP-SNP interactive effects. The first step applied a computationally efficient algorithm, a difference test of odds ratios in hypertensive group and normotensive group [30], to scan all possible pair-wise SNP-SNP interactive effects. A large significance threshold of −log_10_(p)>8 was considered to control false positive. The second step further verified the identified interactive effects using exact conditional logistic regressions [23]–[25], which was computationally intensive but accurate even for sparse data. In general, the two-step procedure helps to reduce but may not exclude all false positive due to 4.2 billion of tests were conducted in the first step. A verification of the identified interactive effects in a CMAS becomes critically important.

An important issue is to consider population admixture/stratification, which may cause spurious association, in population-based case-control studies. This study analyzed Han Chinese samples in the Taiwanese population. In addition to 2 to 3% aborigine people and foreign residents, the Taiwanese population consists of the three major Han Chinese subgroups: Minnan (70%), Hakka (13%) and Mainlanders (14%). Previous studies showed that the high homogeneity of genetic distribution and linkage disequilibrium structure among the three Han Chinese subgroups relative to the Caucasian population. An impact of population admixture on the results of case-control association studies for the Taiwan Han Chinese population is small [35]–[37]. Our population admixture analyses using genome-wide SNP markers also suggested the same conclusion.

This study is the first two-stage GWAS for YOH in the Han Chinese population. We successfully identified novel genetic variants associated with YOH as well as those with interactive effects by applying a p-value combination analysis and a pair-wise interaction analysis. At the first stage, GWAS identified two significant SNP sets that were located in gene regions by using conditional logistic regressions in conjunction with a p-value combination test. SNP quartet rs9308945-rs6711736-rs6729869-rs10495809 located on chromosome 2p22.3 was re-confirmed in the second-stage analysis. Several studies found suggestive linkage signals on chromosome 2p. In particular, HERITAGE Family Study [38] and NHLBI Family Blood Pressure Program [39] showed suggestive evidence at 2p22.3 for African Americans. The quartet was 219 kb, 322 kb, 457 kb, and 495 kb downstream of *LOC344371* (hypothetical gene), *MYADML* (pseudo gene), *FAM98A* (hypothetical protein), and *RASGRP3*, respectively. RAS Guanyl Nucleotide-releasing protein 3 is a member of the RAS subfamily of GTPases which functions in signal transduction as GTP/GDP-regulated switches and serves as RAS activators. Inhibition of RAS-GTPase signaling by chronic FPTIII treatment in streptozotocin-induced diabetic spontaneously hypertensive rats could ameliorate abnormal vascular responsiveness to endothelin-1, angiotensin II in isolated carotid artery. Moderate reduction on mean arterial blood pressure was also observed. Whether it is *RASGRP3* that involves in the YOH development and how the discovered locus on 2p22.3 is connected await for further functional studies.

Furthermore, GWAS also identified eight interactive SNP pairs that passed SNP quality examination and were located in gene regions. SNP pair rs1886985-rs6129969, which showed a significant pair-wise interaction in associating with YOH, was re-confirmed in the second-stage analysis. SNP rs1886985 is located in *IMPG1* on chromosome 6, and rs6129969 and rs2206416 are located in an intergenic region on chromosome 20. *IMPG1*, which is located on 6q14.2-q15, encodes interphotoreceptor matrix proteoglycan 1, which may participate in retinal adhesion and in maintaining photoreceptor viability [40]. *IMPG1* contains 17 exons, including an alternatively spliced exon 2 [41]. A Leu579Pro mutation in *IMPG1* may have a causal role in benign concentric annular macular dystrophy based on a linkage study of a large Dutch family [42]. No association has previously been found between *IMPG1* and hypertension or related traits. Gene *IMPG1* has rat homologue. The gene ID is 66014 for *IMPG1* with respective to *Rattus norvegicus*.

We carried out a preliminary gene expression study comparing pooled samples from three SHR and from three WKY rats at 4, 12, 26 and 38 weeks of age [43]. The use of SHR and WKY rats was approved by the Academia Sinica Institutional Animal Care and Utilization Committee. cDNA was hybridized with NimbleChip Array (Roche NimbleGen, Madison, WI, USA) and analyzed with Gene Spring 7.3.1 (Agilent Technologies, Palo Alto, CA, USA). The *IMPG1* mRNA in SHR rats was 3.12-fold higher than that of WKY rats at 4 weeks prior to the blood pressure elevation in SHR rats, but not at other time points suggesting its potential involvement in the early phase of hypertension development. Proteoglycans are a major component of the animal extracellular matrix and may be present in many adult tissues including blood vessels [44] and nervous tissue [45]. It is capable of binding cations and its synthesis is affected by cation status [46]. *IMPG1* expression may modify blood vessel structure and affect the activity and stability of proteins and signaling molecules within the matrix. In-depth functional studies are, however, required to examine how this gene interactively exerts its effects in humans.

In this study, we applied significance criteria −log_10_(pFDR)>3 and −log_10_(p)>8 for the genome-wide association/interaction tests to reduce false-positive and false-discovery rates. In addition, only significant SNPs, haplotypes and interactions that were located in the region of known genes with potential biological implications were further verified in the CMAS. The use of such criteria may have resulted in a failure to identify biologically relevant SNPs with a relatively small effect. Therefore, it may be worthwhile to examine more SNPs in CMAS in the future by altering the criteria of significance in the GWAS. For example, we found two SNPs with −log_10_(p)>5, neither of which was significant if an FDR correction was considered in single-locus association. The first SNP, rs1010330 on chromosome 2, had a −log_10_(p) of 5.5229 and 6.0000 for CLR-NOMINAL and CLR-ORDINAL analyses, respectively. There have been no genes identified near this SNP. The second SNP, rs864603 on chromosome 21, had a −log_10_(p) of 5.2218 and 5.5229 for CLR-NOMINAL and CLR-ORDINAL analyses, respectively. SNP rs864603 is located in gene *SYNJ1*, and the 100-kb flanking region also contains *C21orf59* and *OR7E23P*. These two SNPs may be investigated further using additional samples and denser SNP chips. The second example includes the 20 significant SNP triplets identified in at least one of the GWAS analyses (CLR-NOMINAL and CLR-ORDINAL). Only the three SNP triplets that were significant in both types of analyses were verified. It would also be worthwhile to verify the other SNP triplets that were significant in only one kind of analysis, since each type of regression model has its unique genetic meaning.

This study can be improved by recruiting more samples and using denser SNP chips. Using CaTs [19], we provided an approximate estimate of power for our two-stage association study. In general, the two-stage association study had sufficiently high power to detect SNP loci with a large main effect (e.g., genotype relative risk >2). However, our association study had reduced power because of the relatively small sample size of 175 case-control pairs in the first stage GWAS. Some small-effect YOH loci may have been missed despite of the higher sample size/power in the second stage CMAS. More samples should be recruited for our next genome-wide scan. However, due to the reasonable sample size for the CMAS, the findings on the YOH-associated genes should be real. On the other hand, this genome-wide study was conducted based on data from the Affymetrix Human Mapping 100K Set. The results can be improved upon by using denser SNP chips, such as the Affymetrix 500K/Array6.0 gene chips and Illumina 550K/1M bead chips. We anticipate that more potential loci may emerge when a denser chip is used with a larger number of samples.

YOH is a common disorder with a complex disease etiology that involves biologically important variants with minor to moderate effects. A single-locus association test is limited in its power to discover this type of common disease variant. This phenomenon was also observed in our study, where no single SNPs were identified as significantly important variants associated with YOH. To overcome the difficulty of identifying common variants that are associated with YOH, we performed several multilocus association tests and interaction tests and successfully identified some novel YOH disease genes. The success of this study highlights the importance of using sophisticated statistical association methods in addition to traditional single-locus association tests. By using different methodological constructions, the multilocus association tests and interaction analyses were able to detect genes involved in joint and interactive models, respectively. The employed p-value combination method (i.e., truncated product p values) utilizes the accumulated significant association signals from proximal SNP markers, which is useful for genetically mapping gene regions containing multiple SNPs that are actually associated with YOH. Haplotype analysis relies on linkage disequilibrium and is more powerful for discovering gene regions containing specific YOH-related haplotypes or haplotype combinations. Interaction analysis is especially designed for detecting combinations of SNPs that act together through pathways or in regulated mechanisms, even though they are located in remote regions or on different chromosomes. These methods provide complementary information for gene mapping. More methods that handle genetic heterogeneity and complexity should be developed to utilize genomic information fully for gene mapping.

Replication of the novel findings is an important issue in association studies. Our CMAS has successfully replicated several SNP loci identified by our GWAS based on the same Taiwanese population. In future, further confirming the results from independent populations helps strengthen the credibility of our findings scientifically [47]. We are working on the replicating studies from independent populations by the following two ways. First, we have collaborated with a Hong Kong young hypertension study group to replicate our results. The study will help replicate our findings from a same Han Chinese population with various life styles and environment. Second, we are applying the data of hypertension GWAS of the Wellcome Trust Case Control Consortium. The study will help validate our results in a non-Han Chinese population. In addition to replication studies, we are also conducting a microarray gene expression study to examine the mRNA-level transcriptional difference of the identified genes.

## Supporting Information

Figure S1 Results of genome-wide single-locus association analysis. The y axis denotes −log_10_(pFDR) and the x axis denotes cumulative physical positions on autosomes. Different colors and symbols show the results on different chromosomes. (A) Results based on a CLR-NOMINAL analysis. (B) Results based on a CLR-ORDINAL analysis.
(0.28 MB TIF)

Figure S2 Results of genome-wide haplotype trend regression analysis. The y axis denotes −log_10_(pFDR) and the x axis denotes cumulative physical positions on autosomes. Results of haplotype trend regression analyses for different minimum haplotype frequencies are showed: (A) <0.01, (B) <0.05 and (C) <0.10.
(0.08 MB TIF)

Figure S3 LD structures of SNPs rs9308945, rs6711736, rs6729869 and rs10495809 in control, case and combined groups. The LD block contains four SNPs. The pairwise D′ and frequencies of major haplotypes are shown: (A) LD structure in control group, case group and combined group, (B) haplotype frequencies in control group, case group and combined group.
(0.11 MB TIF)
