# Supplementary figures and images for: Genome-Wide Association Study of Young-Onset Hypertension in the Han Chinese Population of Taiwan
Source: PLoS One. 2009 May 7;4(5):e5459. doi: 10.1371/journal.pone.0005459 (PMC2674219; doi:10.1371/journal.pone.0005459)

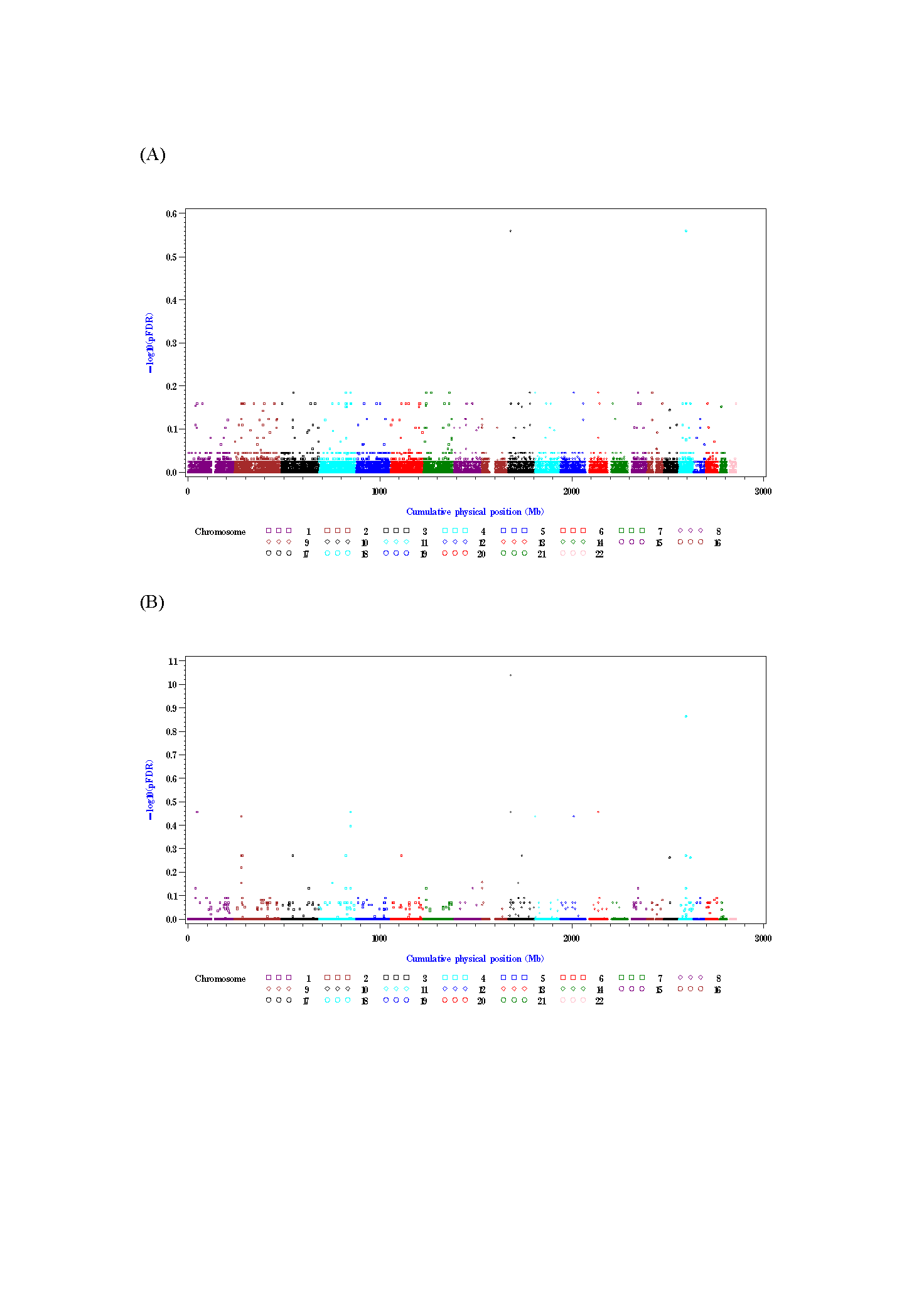

Supplement: Figure S1 — Results of genome-wide single-locus association analysis. The y axis denotes −log10(pFDR) and the x axis denotes cumulative physical positions on autosomes. Different colors and symbols show the results on different chromosomes. (A) Results based on a CLR-NOMINAL analysis. (B) Results based on a CLR-ORDINAL analysis. (0.28 MB TIF) [file pone.0005459.s001.tif]

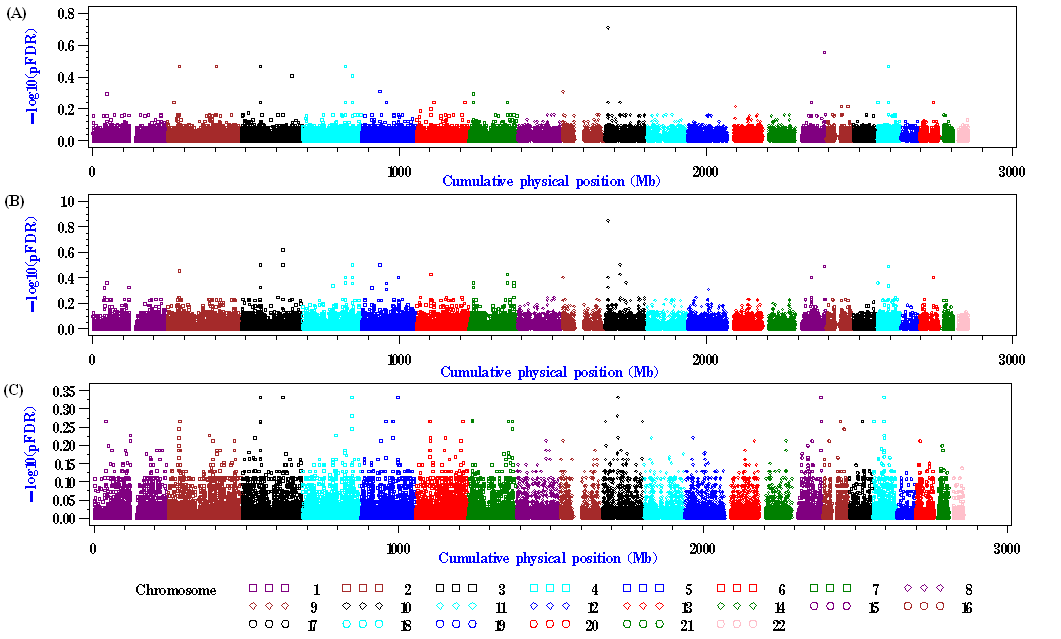

Supplement: Figure S2 — Results of genome-wide haplotype trend regression analysis. The y axis denotes −log10(pFDR) and the x axis denotes cumulative physical positions on autosomes. Results of haplotype trend regression analyses for different minimum haplotype frequencies are showed: (A) <0.01, (B) <0.05 and (C) <0.10. (0.08 MB TIF) [file pone.0005459.s002.tif]

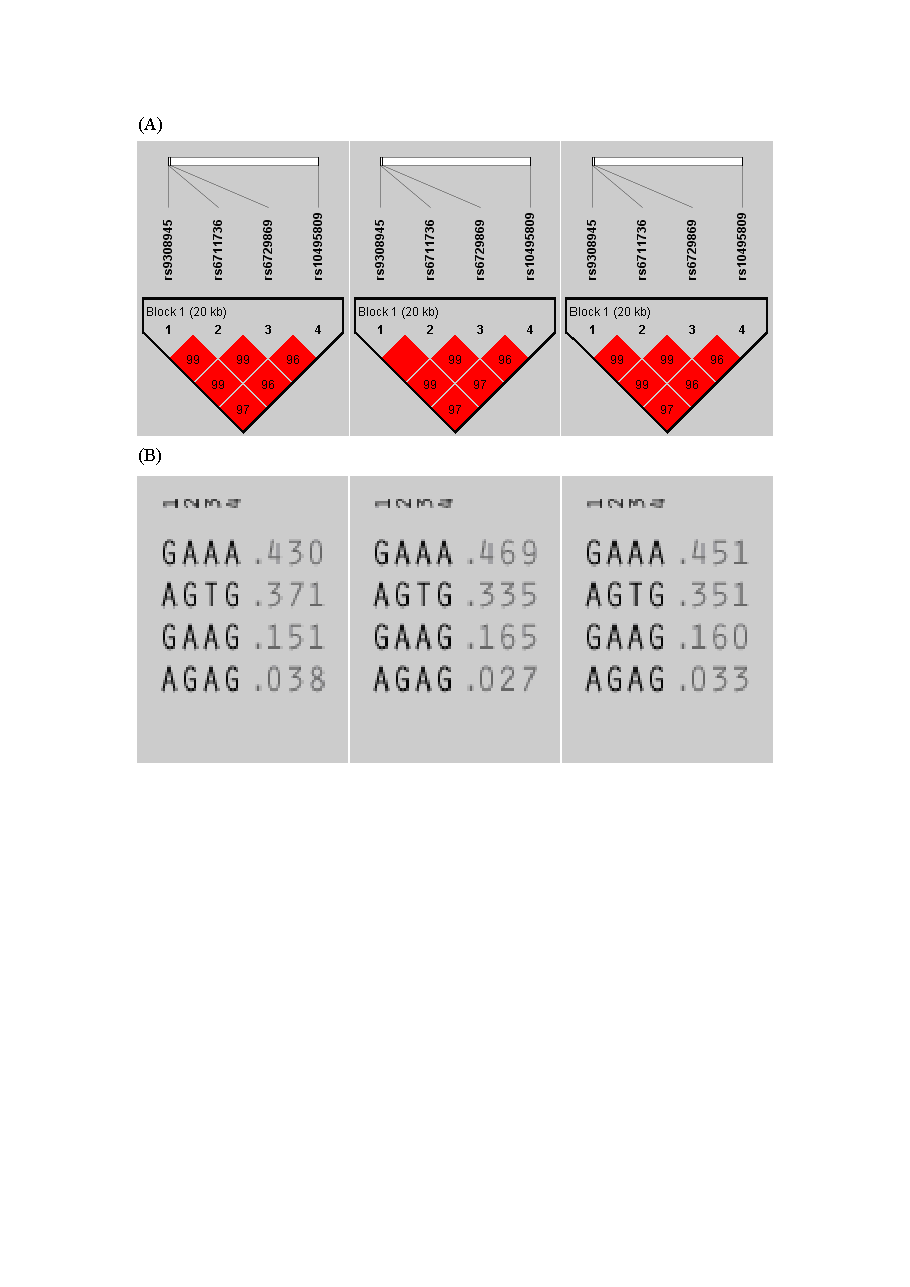

Supplement: Figure S3 — LD structures of SNPs rs9308945, rs6711736, rs6729869 and rs10495809 in control, case and combined groups. The LD block contains four SNPs. The pairwise D′ and frequencies of major haplotypes are shown: (A) LD structure in control group, case group and combined group, (B) haplotype frequencies in control group, case group and combined group. (0.11 MB TIF) [file pone.0005459.s003.tif]
